# Effects of dietary chito-oligosaccharide and β-glucan on the water quality and gut microbiota, intestinal morphology, immune response, and meat quality of Chinese soft-shell turtle (*Pelodiscus sinensis*)

**DOI:** 10.3389/fimmu.2023.1266997

**Published:** 2023-10-26

**Authors:** Hao Fu, Ming Qi, Qingman Yang, Ming Li, Gaohua Yao, Weishao Bu, Tianlun Zheng, Xionge Pi

**Affiliations:** ^1^ Institute of Plant Protection and Microbiology, Zhejiang Academy of Agricultural Sciences, Hangzhou, China; ^2^ Zhejiang Fisheries Technical Extension Center, Hangzhou, China; ^3^ Shaoxing Fisheries Technical Extension Center, Shaoxing, China; ^4^ Jinhua Fisheries Technical Extension Center, Jinhua, China; ^5^ Qingjiang Professional Cooperative for Ecological Farming Turtles, Lishui, China; ^6^ Institute of Rural Development, Zhejiang Academy of Agricultural Sciences, Hangzhou, China

**Keywords:** chito-oligosaccharide, β-glucan, gut microbiota, intestinal morphology, immune response, meat quality, *Pelodiscus sinensis*

## Abstract

Chito-oligosaccharides (COS) and β-glucan are gradually being applied in aquaculture as antioxidants and immunomodulators. However, this study examined the effects of dietary supplementation of COS and β-glucan on the water quality, gut microbiota, intestinal morphology, non-specific immunity, and meat quality of Chinese soft-shell turtle. To investigate the possible mechanisms, 3-year-old turtles were fed basal diet (CK group) and 0.1%, 0.5%, and 1% COS or β-glucan supplemented diet for 4 weeks. Colon, liver, blood and muscle tissues, colon contents, water and sediment of paddy field samples were collected and analyzed after feeding 2 and 4 weeks. The results indicated that COS and β-glucan altered microbial community composition and diversity in Chinese soft-shell turtles. The relative abundance of *Cellulosilyticum*, *Helicobacter* and *Solibacillus* were increased after feeding COS, while *Romboutsia*, *Akkermansia* and *Paraclostridium* were increased after feeding β-glucan, whereas *Cetobacterium*, *Vibrio* and *Edwardsiella* were enriched in the control group. Furthermore, colon morphology analysis revealed that COS and β-glucan improved the length and number of intestinal villi, and the effect of 0.5% β-glucan was more obvious. Both β-glucan and COS significantly improved liver and serum lysozyme activity and antibacterial capacity. COS significantly increased the total antioxidant capacity in the liver. Further, 0.1% β-glucan significantly increased the activity of hepatic alkaline phosphatase, which closely related to the bacteria involved in lipid metabolism. Moreover, dietary supplementation with 1% COS and 1% β-glucan significantly enhanced the content of total amino acids, especially umami amino acids, in muscle tissue, with β-glucan exerting a stronger effect than COS. Additionally, these two prebiotics promoted the quality of culture water in paddy fields and reshaped the bacterial community composition of aquaculture environment. All these phenotypic changes were closely associated with the gut microbes regulated by these two prebiotics. In summary, the findings suggest that dietary supplementation with COS and β-glucan in *Pelodiscus sinensis* could modulate the gut microbiota, improve intestinal morphology, enhance non-specific immunity and antioxidant capacity of liver and serum, increase meat quality, and improve the culture water environment. This study provides new insights and a comprehensive understanding of the positive effects of COS and β-glucan on *Pelodiscus sinensis*.

## Introduction

The Chinese soft-shell turtle (*Pelodiscus sinensis*, hereinafter referred to as “turtle”) is an important economic species in aquaculture production owing to its high nutritional value and unique taste (1, 2). With the depletion of their numbers in the wild, soft-shell turtles are now being produced through a variety of breeding modes (such as greenhouse breeding, pond breeding, and ecological breeding). Among these strategies, greenhouse farming has emerged as an important mode of turtle breeding in recent decades, but it requires a large amount of artificial aquafeed. Moreover, drug treatment to promote growth and prevent diseases is required and supposed to in the culturing process, which causes serious water pollution and leads to drug residues in turtle meat, such as quinoxalines (3, 4). In contrast, the cultivation of soft-shell turtles in paddy fields represents a mode of ecological culture and prevents the disadvantages associated with greenhouse farming. Various excess resources present in the paddy field can be utilized by the turtles, and the turtles can in turn perform weeding, thinning, and pest control activities in the paddy field (5). However, this form of turtle breeding also has some drawbacks, such as challenges in disease prevention and promoting turtle growth. Recently, prebiotics have shown efficacy in disease prevention and treatment in aquaculture. Several types of prebiotics, including xylooligosaccharide and galactooligosaccharide, have been used as antibiotic alternatives to promote intestinal health and prevent disease by selectively promoting the growth of beneficial bacteria (6).

Chito-oligosaccharide (COS), a natural alkaline polymer of glucosamine, has been used as a feed additive for aquatic animals, adjuvant for vaccines, and water preservation agent for aquatic products owing to its useful functions in regulating immunity and promoting growth and antioxidant responses in aquatic animals (7, 8). Dietary COS improved the growth performance, body composition, digestive enzyme activity, and antioxidant responses of the loach *Paramisgurnus dabryanus* (9). Moreover, strong antibody reactions and cross-protection against *Vibrio alginolyticus* and *Vibrio harveyi* were observed in turbots vaccinated with inactivated vaccine and COS (10). Jia et al. found that COS can inhibit the growth of *Pseudomonas*, *Aeromonas*, and *Shewanella* in silver carp fillets, improving their meat quality during cold storage (11). Another agent that has been used as an immunomodulator and antioxidant in the aquaculture industry is β-glucan, a type of complex polysaccharide (12, 13). In recent studies, both COS and β-glucan were tested as substitutes for in-feed antibiotics and were found to modulate the gut microbiome and immune responses, thereby improving gastrointestinal conditions and growth performance (14).

Previous studies have reported that dietary supplementation of β-glucan could improve the immunity and antioxidant indexes of turtles (15), but systematic studies are still lacking, especially on the rice-turtle cultivation mode. Hence, the present study was designed to investigate the effects of different levels of dietary COS and β-glucan on the environmental water quality and gut microbiota, intestinal morphology, non-specific immunity (liver and serum), and meat quality of Chinese soft-shell turtles in the paddy fields. Further, we revealed the possible mechanisms underlying the improvements in immune responses, meat quality, and growth induced by these prebiotics. This work provides a more comprehensive understanding of the beneficial effects of COS and β-glucan on turtles and promotes the application of prebiotics in aquaculture.

## Materials and methods

### Experimental design and sampling

**Figure 1:**
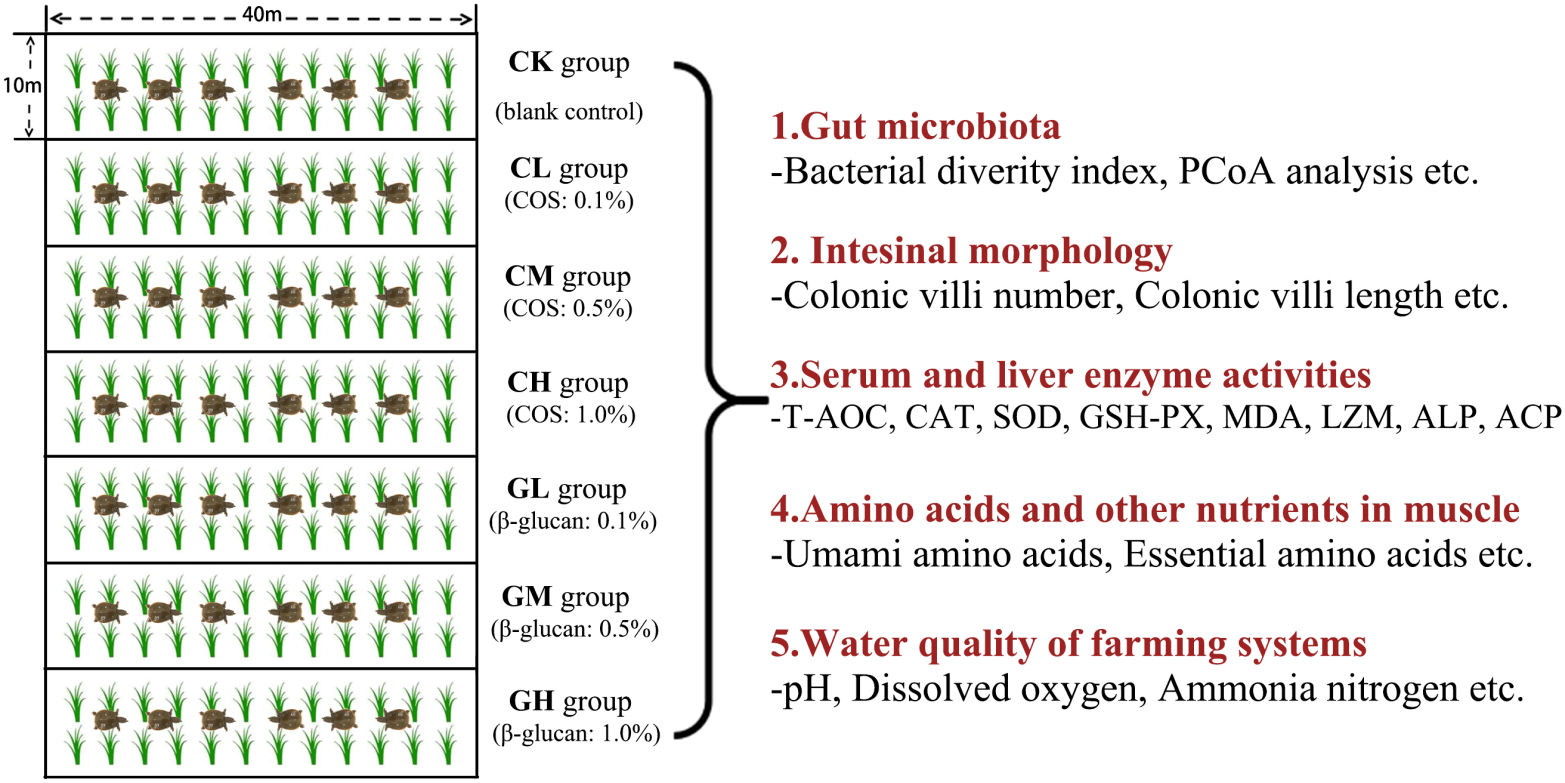
Flow chart of experimental design.

The experiment was conducted in seven paddy fields (measuring about 40 m × 10 m, each paddy field was averagely divided into three small fields). Three-year-old Chinese soft-shell turtles (mean initial body weight: 1 ± 0.2 kg, mean carapace length: 20.2 cm) were randomly divided into seven groups (six experimental diet groups + one control group) in these seven paddy fields and maintained on the indicated diet for 4 weeks during September 2022 in Lishui City, Zhejiang province. The six types of experimental diets were formulated by adding different doses of COS (0.1%, CL; 0.5%, CM; and 1%, CH) or β-glucan (0.1%, GL; 0.5%, GM; and 1%, GH) to the basal diet. The control group only received a basal diet. COS and β-glucan were purchased from Zhejiang Golden-Shell Pharmaceutical Co. Ltd. and Angel Yeast Co. Ltd., respectively. The diet formulation and its proximate composition of the basal feed are shown in **Table 1**. The flow chart of the experimental design is shown in **Figure 1**.

**Table 1:** Diet formulation and proximate composition of the basal feed (dry matter basis, %).

Ingredients	Percentage of ingredients (%)
Crude protein (%, ≥)	46.0
Crude lipid (%, ≥)	5.0
Crude fiber (%, ≤)	4.0
Crude ash (%, ≤)	17.0
Calcium (%, ≤)	2.0-5.0
Phosphorus (%, ≤)	1.0-3.0
Lysine (%, ≥)	2.2
Moisture (%, ≤)	10.0

Providing by Yixiang Biotechnology Co. Ltd. (Huzhou, Zhejiang province, China).

The turtles were fed twice a day (8:30, 17:30). Two (Time point 1, T1) and 4 weeks (Time point 2, T2) after prebiotic feeding were selected as sampling time points. Six turtles were randomly sampled from each group (two turtles were randomly selected from each one small paddy field) at T1 and T2, and their body weight was measured. Blood samples were collected from the jugular veins and then centrifuged (3500 ×*g*) for 15 min at 4°C to obtain serum samples. Hind leg muscle tissue, liver tissue, intestine tissue, and colon contents were collected and frozen in liquid nitrogen. All samples were stored at -80°C until use.

### 16S rRNA gene sequencing for gut and water microbiota analysis

Colon contents were collected from six turtles in each group at two time points. Water and sediment samples were also collected from the seven paddy fields when the experiment was completed and established enrichment of microorganisms by centrifugation. DNA was extracted from colon content samples, water and sediment samples using the DNeasy PowerSoil Pro Kit (Qiagen, USA) according to the user manual. The concentration and quality of extracted DNA were checked on a 1% agarose gel and measured using NanoDrop 2000 (Thermo Scientific, USA). The V3-V4 hypervariable region of the bacterial 16S rRNA gene was amplified using the following primer pairs: 338F- ACTCCTACGGGAGGCAGCAG; 806R- GGACTACHVGGGTWTCTAAT. The PCR reaction mixture including 10 μL 5 × Fast reaction buffer, 1.5 μL 2.5 mM dNTPs, 1.5 μL each primer (10 μM), 0.2 μL Fast DNA polymerase, 50 ng of template DNA, and ddH_2_O to a final volume of 50 µL. PCR amplification cycling conditions were as follows: initial denaturation at 95 °C for 5 min, followed by 30 cycles of denaturing at 95 °C for 1 min, annealing at 60 °C for 1 min and extension at 72 °C for 1 min, and single extension at 72 °C for 7 min, and end at 4 °C. After purification, PCR products were subjected to paired-end sequencing on the Illumina MiSeq PE300 platform (Illumina, USA). All 16S rRNA gene sequencing data were submitted to the NCBI database (accession number PRJNA974559).

Raw 16S rRNA gene sequencing reads were demultiplexed, quality-filtered using fastp (v0.20.0) (16), and merged into tags using FLASH (v1.2.7) (17). Chimeric sequences were filtered with the Vsearch software (v2.3.4) (18). The feature table and sequence were obtained after dereplication with DADA2 (19). A Naive Bayes classifier was used to annotate the amplicon sequence variant (ASV) table according to the SILVA database (v138) for each representative sequence (20). Then, feature abundance was normalized based on the relative abundance in each sample. The ASV table was leveled according to the minimum number of sequences in all samples. α-diversity indexes, including the Chao1 and Shannon indexes, were calculated to evaluate microbial community complexity using QIIME2 (21). β-diversity was analyzed to investigate the differences within and between groups based on principal coordinates analysis (PCoA) with the R vegan package (22). Further, microbial functional prediction was achieved using PICRUSt2 (23). Functional pathway annotation was performed based on the Kyoto Encyclopedia of Genes and Genomes (KEGG) database (24). All data processing and analyses were conducted using OmicShare Tools at https://www.omicshare.com/tools.

### Determination of antioxidant and immune parameters in serum and liver tissue

Serum and liver samples were thawed on ice. Then, the liver samples were homogenized for subsequent analysis. Five indicators of antioxidant action in the serum and liver were examined using commercial kits (Nanjing Jiancheng Bioengineering Institute, China) according to the manufacturer’s instructions. These were as follows: total antioxidant capacity (T-AOC) and catalase (CAT), superoxide dismutase (SOD), glutathione peroxidase (GSH-Px), and malondialdehyde (MDA) levels. T-AOC activity was measured using colorimetry. CAT activity was measured using the visible light method. SOD activity was measured using the WST-1 method. GSH-PX activity was measured using a colorimetric method. Finally, MDA content was measured using the thiobarbituric acid (TBA) method.

In addition, three indicators of non-specific immunity were also determined. Lysozyme (LZM), alkaline phosphatase (ALP), and acid phosphatase (ACP) levels in the serum and liver were measured using commercial kits (Nanjing Jiancheng Bioengineering Institute, China) according to the manufacturer’s instructions. LZM activity was measured using turbidimetry. Further, ALP and ACP activity were both measured using a microenzyme labeling method.

All reactions were performed in 96-well plates, and the absorbance was read using a microplate reader (Thermo Scientific™ 5580, China).

### Amino acid and proximate compositions of muscle

First, 50 g of muscle tissue was isolated from the two hind legs to determine its amino acid composition and proximate composition, as described previously (25, 26). Briefly, the muscle tissue was ground into powder after freezing in liquid nitrogen. Then, 40 mg freeze-dried muscle powder and 10 mL of 6 mol/L hydrochloric acid were added to a 50-mL ampoule, which was then placed in a constant temperature drying oven for 24 h to enable hydrolysis. The hydrolysate was diluted with deionized water, transferred to a rotary evaporator flask, and evaporated to dryness at 60°C. The dehydrated product was washed with 0.02 mol/L hydrochloric acid and transferred to another volumetric flask. Then, a 3 mL hydrolysate sample and a standard amino acid solution were added to an automated sample injection bottle. The content of amino acids in the muscle tissue was determined by the automatic amino acid analyzer (Hitachi Model L8900, Japan). The moisture content was determined based on the 105 ± 2°C oven drying method. The crude ash content was determined by carbonization with the 525 ± 25°C furnace carbonizing method. The crude fat content was determined using the Soxhlet extraction method with petroleum ether as the solvent.

### Intestinal morphology

Colon tissue was washed with phosphate-buffered saline (PBS) and quickly fixed in 4% paraformaldehyde. Paraffin embedding was performed 24 h later. Sections were sliced longitudinally to 4-µm thickness, hydrated, and stained with hematoxylin-eosin (H&E) using an automatic dehydrator. Histomorphology was observed using a digital pathology scanner (Kfbio, China) and a light microscope (Nikon, Japan).

### Water quality measurement

Five indicators of water quality were measured after the experiment was completed. Water samples were collected from each paddy field in triplicate. pH was measured on-site at the time of sample collection using an Oakton Acorn pH meter (Sigma-Aldrich, Germany) (27). Dissolved oxygen levels were determined using a Pro-DSS multiparameter meter (YSI, US) (28). The total ammonia nitrogen content was measured using Solorzano’s indophenol method (29). Nitrite levels were determined using an azo dye at a wavelength of 543 nm after processing with sulfanilamide hydrochloride and N-(1-naphthy1)-ethylenediamine dihydrochloride (30). The ammonia nitrogen and nitrite levels were determined using an Epoch^™^ spectrophotometer (Biotek US). The free sulfides were measured using the methylene blue method with a PowerWave^™^ spectrophotometer microplate reader (Biotek, US) (31).

### Statistical analysis

The results of the α-diversity indexes, nutrient contents, and enzyme activities were expressed as the means ± standard deviations (SDs) and analyzed using one-way analysis of variance (ANOVA) followed by Tukey’s multiple comparison test between multiple groups. GraphPad Prism 8.0.1 was used for data analysis and figure creation. The correlations among enzyme activities, nutrient contents, and microbial taxa were analyzed using Spearman’s rank correlation analysis. Statistical significance was set at *P* < 0.05.

## Results

### Composition and diversity of gut microbiota

**Figure 2:**
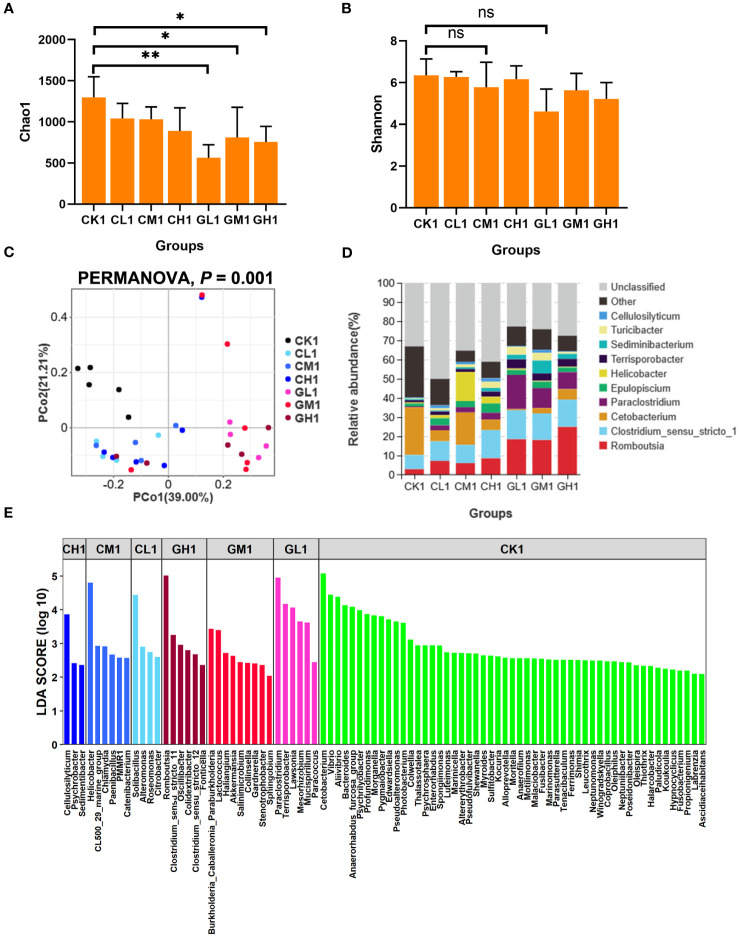
Comparison of the diversity and bacterial composition of the gut microbiota at T1 based on 16S rRNA gene sequencing data. **(A)** Chao1 index. **(B)** Shannon index. **(C)** Principal coordinate analysis (PCoA) of the microbial composition of various groups based on weighted UniFrac distances. **(D)** Relative abundance of the top 10 bacterial genera in each group. **(E)** Differential bacterial genera identified among the seven groups using LEfSe analysis. LDA score > 2. LDA, linear discriminant analysis. PERMANOVA, permutational multivariate analysis of variance. ns, no significance. one-way ANOVA, *P < 0.05 and **P < 0.01.

The α-diversity of the gut microbiota was significantly different between the CK group and the COS and β-glucan groups at the two time points (**Figures 2A, B**, **S1A, B**). Notably, the Chao1 index of the COS group was significantly decreased at T1 and then increased at T2 (**Figures 2A**, **S1A**). The Chao1 index of the COS group was also significantly higher than that of the β-glucan and CK groups (**Figure S1A**). PCoA revealed that the three groups showed significant differences in β-diversity at T1 (PERMANOVA, *P* = 0.001) (**Figure 2C**), but the differences were fewer after 4 weeks of feeding (PERMANOVA, *P* = 0.01) (**Figure S1C**). The genus-level bacterial composition of the gut microbiota was different among the COS, β-glucan, and CK groups (**Figures 2D, E**, **S1D, E**). A total of 78 genera were enriched in different diet groups after 2 weeks. Among them, *Romboutsia* and *Paraclostridium* showed a higher abundance in both the COS and β-glucan groups than in the CK group, and higher amounts of COS or β-glucan were more effective in increasing the abundance of these two genera. Meanwhile, *Cetobacterium* and 52 other genera were enriched in the CK group. Moreover, the COS and β-glucan groups also showed differences in genus-level enrichment. For example, *Cellulosilyticum*, *Helicobacter*, and *Solibacillus* were more enriched in the COS group, while *Romboutsia*, *Paraclostridium*, and *Terrisporobacter* were more abundant in the β-glucan group (**Figures 2D, E**; **Supplementary Table S1**). At T2, the microbial community enrichment profile was similar to that at T1 (**Figures S1D, E**; **Supplementary Table S1**). All results suggested that the addition of COS or β-glucan to the diet could change the composition of the gut microbiome in turtles and enable the differential enrichment of specific bacteria.

### Alteration of intestinal morphology

**Figure 3:**
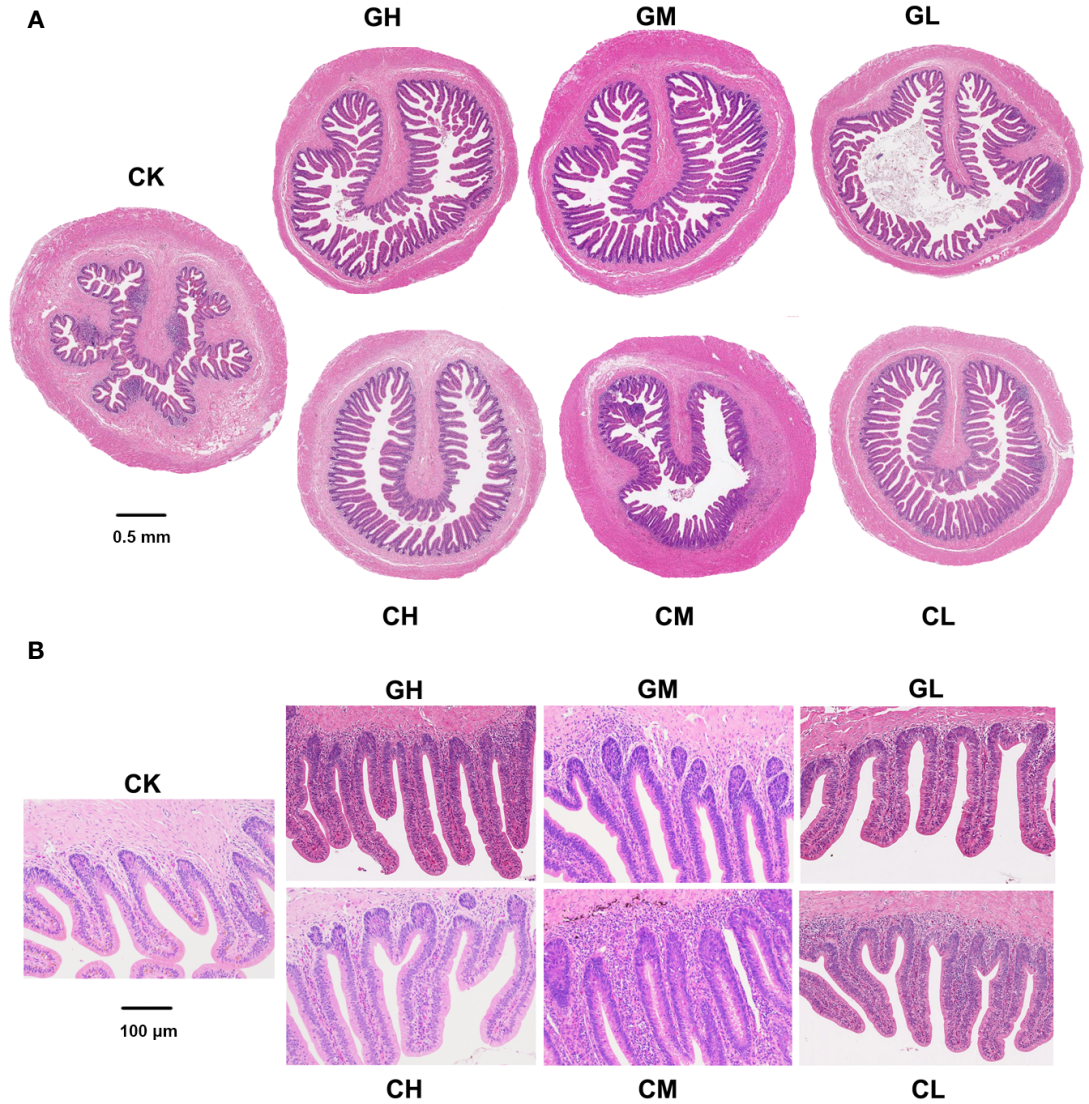
Colon morphology of *P. sinensis* at T1. **(A)** Full scans (×20). **(B)** Partial scans (×100).

The number and length of colonic villi in turtles showed a drastic increase after dietary supplementation with COS and β-glucan (**Figure 3**). The effect of β-glucan was stronger than that of COS and improved over time (**Figure S2**). In fact, the middle concentration (0.5%) of β-glucan provided the best improvement.

### Changes in liver and serum enzyme activities related to antioxidant capacity and immunity

**Figure 4:**
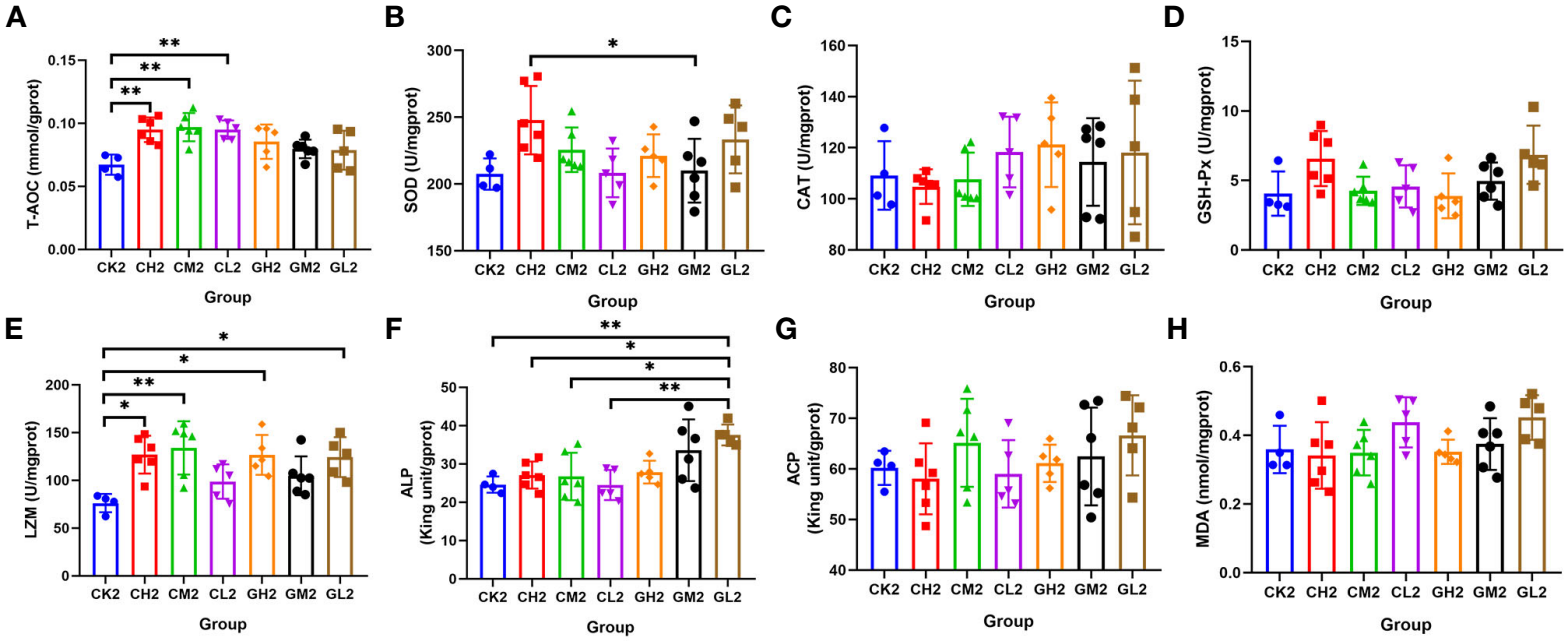
Effects of COS and β-glucan on the activities of liver enzymes related to antioxidant capacity and immunity in *P. sinensis* at T2. **(A)** T-AOC, total antioxidant capacity. **(B)** SOD, superoxide dismutase. **(C)** CAT, catalase. **(D)** GSH-Px, glutathione peroxidase. **(E)** LZM, lysozyme. **(F)** ALP, alkaline phosphatase. **(G)** ACP, acid phosphatase. **(H)** MDA, malondialdehyde. one-way ANOVA, *P < 0.05, **P < 0.01.

**Figure 5:**
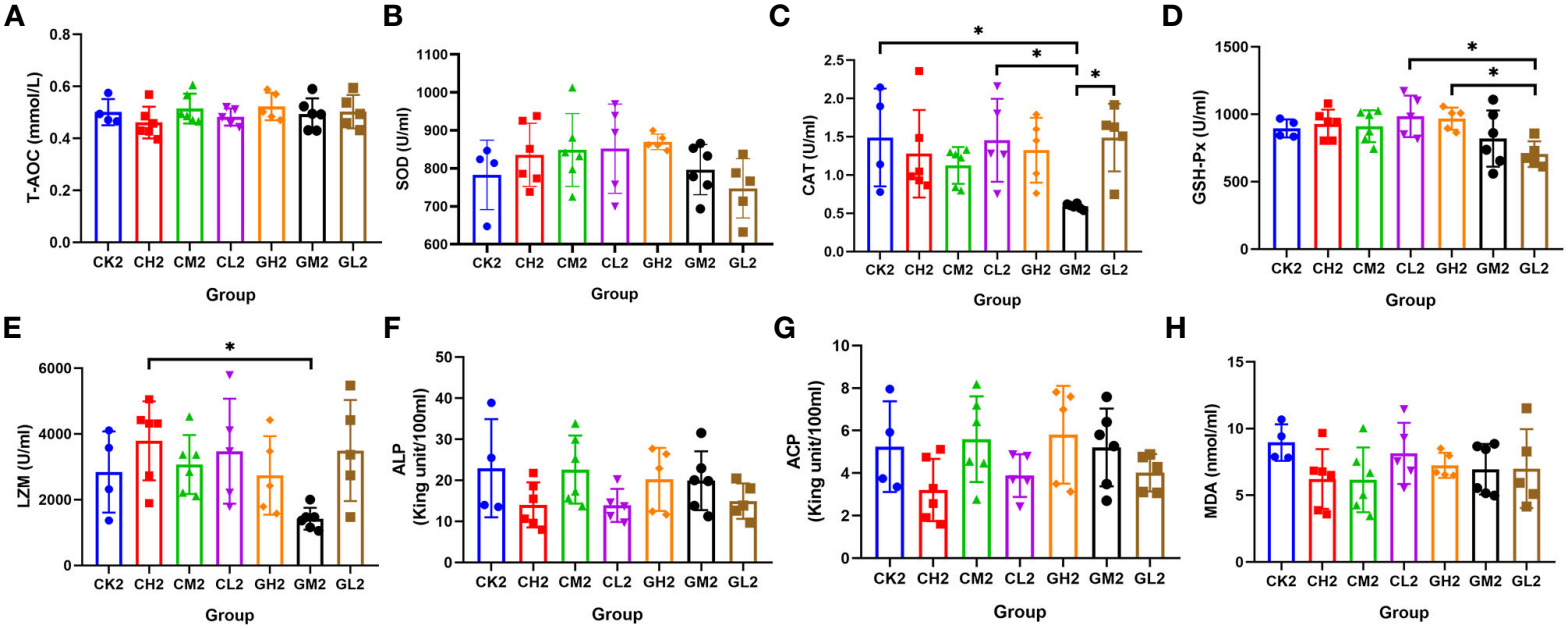
Effects of COS and β-glucan on the activities of serum enzymes related to antioxidant capacity and immunity in *P. sinensis* at T2. **(A)** T-AOC, total antioxidant capacity. **(B)** SOD, superoxide dismutase. **(C)** CAT, catalase. **(D)** GSH-Px, glutathione peroxidase. **(E)** LZM, lysozyme. **(F)** ALP, alkaline phosphatase. **(G)** ACP, acid phosphatase. **(H)** MDA, malondialdehyde. one-way ANOVA, *P < 0.05.

A total of eight serum and hepatic indicators were measured at T2. Liver sample analysis revealed that T-AOC was significantly higher in the COS group than in the CK group, and SOD activity was higher in the COS group than in the β-glucan group (**Figures 4A, B**; **Supplementary Table S2**). This suggested that COS was better at improving antioxidant capacity than β-glucan. Both COS and β-glucan could improve LZM enzyme activity in the liver (**Figure 4E**), indicating their enhancement of antibacterial effects. Additionally, β-glucan induced higher liver ALP activity than COS, and a low concentration (0.1%) of β-glucan showed the best effect (**Figure 4F**). In serum samples, the levels of CAT, GSH-Px, and LZM were higher in turtles receiving COS than in those receiving β-glucan (**Figures 5C–E**; **Supplementary Table S3**), while the levels of other five enzymes had no significant difference between these two supplementary feeding (**Figures 5A, B, F–H**), demonstrating that COS stimulates higher antioxidant and antibacterial activity than β-glucan. Together, the results indicated that these two prebiotics could improve non-specific immunity in turtles, although the mechanisms of their individual effects were slightly different.

The correlations between indicators of non-specific immunity and differential bacterial genera in the turtles were analyzed (**Figure S3**). Many genera enriched in the prebiotics groups showed close correlations with indicators such as LZM and T-AOC. For example, *Romboutsia* was positively related to the level of LZM in both the liver and serum, suggesting that the antimicrobial ability of prebiotics could be attributed to alterations in the gut microbiota. In addition, the genera *Helicobacter* and *Mycoplasma* enriched in the CK group were negatively correlated with the serum levels of SOD. This demonstrated that the decreased antioxidant capacity may be due to a lack of gut microbiota regulation by prebiotics.

### Improvement of amino acid composition and nutrient content of muscle

**Figure 6:**
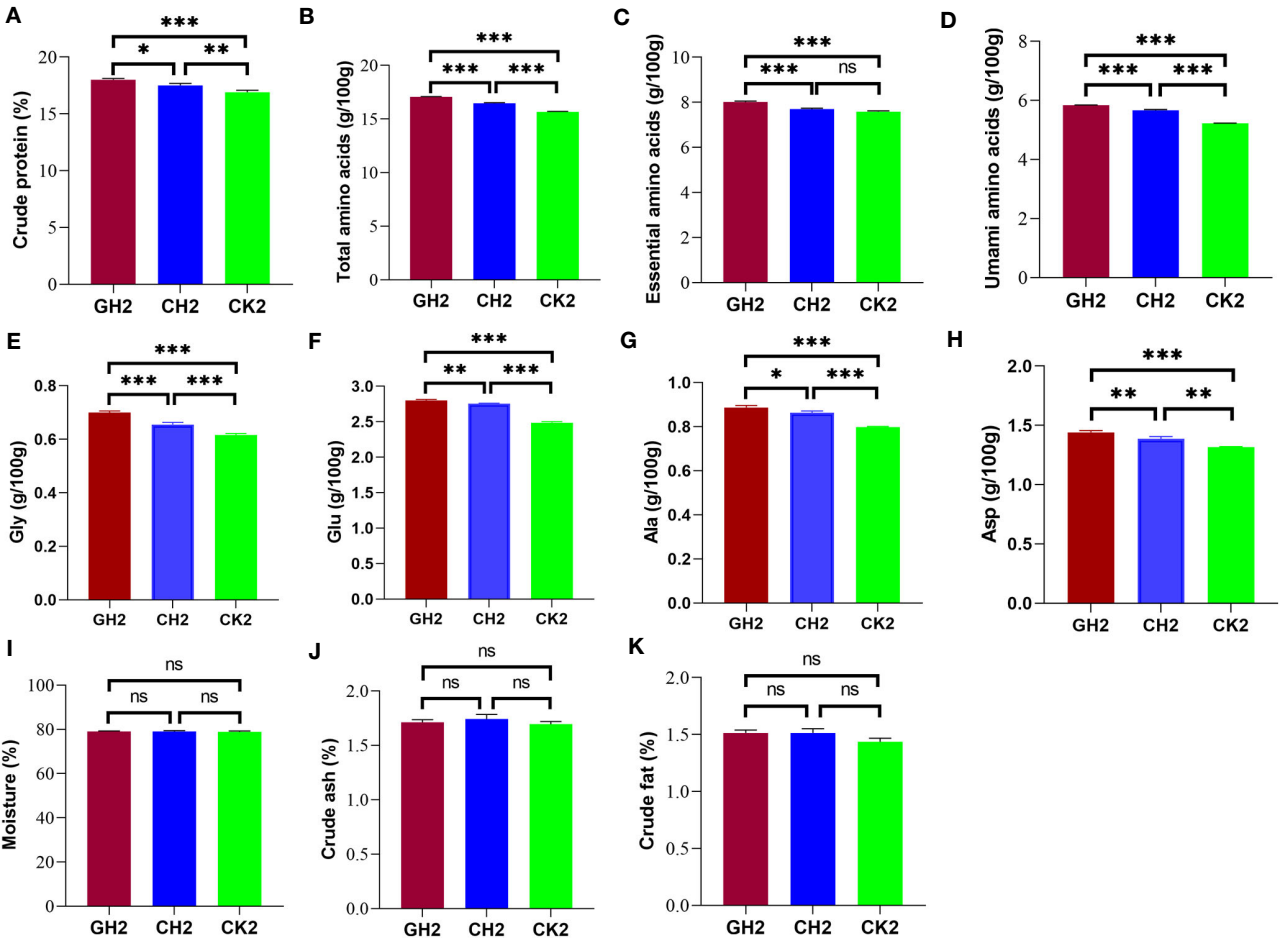
Effects of COS and β-glucan on muscle amino acid and nutrient composition in *P. sinensis* at T2. **(A)** Crude protein. **(B)** Total amino acids. **(C)** Essential amino acids. **(D)** Umami amino acids. **(E)** Gly, glycine. **(F)** Glu, glutamate. **(G)** Ala, alanine. **(H)** Asp, asparagine. **(I)** Moisture. **(J)** Crude ash. **(K)** Crude fat. The content of amino acids was measured per 100 g of muscle, and the proportion of other primary nutrients was compared based on muscle weight. ns, no significance. one-way ANOVA, *P < 0.05, **P < 0.01, and ***P < 0.001.

The levels of crude protein, total amino acids, essential amino acids, and umami amino acids in turtle muscle tissue were significantly higher in the COS and β-glucan groups than in the CK group (**Figures 6A–D**; **Supplementary Table S4**). The positive effect of 1% β-glucan on these parameters was better than that of 1% COS. Moreover, the contents of four kinds of umami amino acids (glycine, glutamate, alanine, and asparagine) were significantly increased by COS and β-glucan supplementation, with 1% β-glucan increasing the levels of umami amino acids by 11.6% when compared with the CK group (**Figures 6E–H**). Meanwhile, there was no significant difference in the muscle contents of moisture, crude ash, and crude fat among the three groups (**Figures 6I–K**). These results indicated that the two prebiotics can improve meat quality, especially the level of umami amino acids.

In addition, the levels of these main nutrients were significantly associated with the abundance of seven differential genera. Among them, *Clostridium sense stricto13* was positively associated with the levels of essential and umami amino acids (**Figure S4**).

### Improvement of water and sediment environments in paddy fields

**Figure 7:**
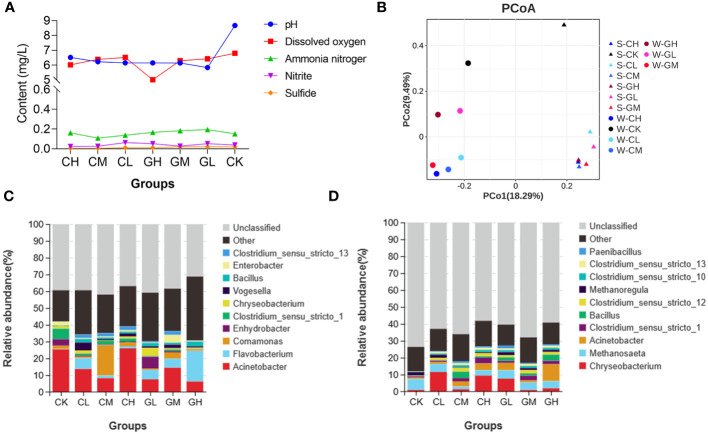
Effects of COS and β-glucan on the quality of culture water and sediment in paddy fields. **(A)** Main indicators of water quality. **(B)** Principal coordinate analysis (PCoA) of the microbial composition of various groups in both water and sediment environment based on weighted UniFrac distances. Bacterial community composition of **(C)** culture water and **(D)** sediment of paddy fields in each group at the genus level. S, sediment; W, water.

Five water quality indicators in the seven paddy fields were measured when the experiment was completed — pH, dissolved oxygen, ammonia nitrogen, nitrite, and sulfide. We found that pH and sulfide levels were lower in the two prebiotics groups than in the CK group (**Figure 7A**; **Supplementary Table S5**). The microbial communities present in the water samples from different experimental paddy fields were different, as did the results of the sediment samples. More important, the microbial community composition was distinctly different between water and sediment samples (**Figure 7B**). Furthermore, the microbial community structures changed at the genus levels after turtles were fed COS and β-glucan in both water and sediment environment (**Figures 7C, D**). At the genus level, the relative abundance of *Flavobacterium* and *Clostridium_sensu_stricto_13* increased, while that of *Clostridium_sensu_stricto_1* decreased after prebiotics treatment in the water of paddy fields. Further, the relative abundance of *Acinetobacter*, *Bacillus* and *Clostridium_sensu_stricto_13* increased, while that of *Methanosaeta* decreased after prebiotics treatment in the sediment of paddy fields. Together, these results demonstrated that the application of prebiotics could improve water quality and change the micro-ecosystem of the water and sediment of paddy fields.

## Discussion

In our study, the gut microbial community structure was significantly different in turtles receiving COS and β-glucan when compared with that in turtles receiving the basal feed. β-glucan decreased the Chao1 index at T1, while COS improved this index at T2. This suggested that these two prebiotics had different functions in modulating the gut microbiome. LEfSe analysis revealed that at the genus level, the relative abundance of *Romboutsia* increased after prebiotic supplementation, particularly in the GH group. However, the relative abundance of *Cetobacterium* decreased after prebiotic addition, but remained enriched in the CK group. *Romboutsia* has been linked to lipid metabolism and obesity-related metabolic abnormalities (32), while *Cetobacterium* plays an important role in carbohydrate utilization and glucose homeostasis in fish (33). The finding thus suggested that COS and β-glucan were biased toward promoting lipid metabolism instead of glucose utilization because the experimental turtles were already adults and fat deposition had started during their late growth stage. Furthermore, some genera such as *Lawsonia* (34), *Vibrio* (35), and *Edwardsiella* (36) that comprise potential pathogens were enriched in the CK group. Analysis of the functional capacity of the gut microbiota revealed that the functions enriched in the prebiotic groups were related to pathways of amino acid, vitamin, glycerolipid, and bile acid metabolism. Meanwhile, the bacterial functions enriched in the CK group involved pathways of pathogen infection and antibiotic biosynthesis, including *Vibrio cholerae* infection, vancomycin group antibiotic biosynthesis, and streptomycin biosynthesis (**Figure S5A**). Moreover, these functional pathways were significantly correlated with the relative abundance of differential bacteria (prebiotics group vs. CK group) (**Figure S5B**). Among them, *Cetobacterium*, *Vibrio*, and *Aliivibrio* were positively associated (*P* < 0.01) with the pathways of pathogen infection and antibiotic biosynthesis, while some genera enriched in the prebiotics group, including *Romboutsia* and *Terrisporobacter*, were negatively correlated with these pathways. Overall, all the results suggested that COS and β-glucan could promote intestinal health, regulate gut microbes, and shape the homeostatic balance of the gut microbiome.

COS and β-glucan not only modulated the gut microbiome, but also altered intestinal morphology, increasing the number and length of colonic villi and the depth of colon crypts. These changes indicate a strengthening of the colon’s epithelial barrier function, which is involved in promoting nutrient absorption and reducing infection (37). β-glucan has been reported to improve the gut microbiota composition in models of metabolic diseases and promote the production of short-chain fatty acids (SCFAs), which improve gut health to alleviate metabolic diseases (38). Early studies have revealed that SCFAs can facilitate the differentiation and proliferation of the intestinal epithelium (39). This could explain how COS and β-glucan improve colonic health at a morphological level and regulate immune homeostasis in the gut and the entire body.

We found that COS and β-glucan promoted non-specific immunity in both the liver and serum. These two prebiotics conferred great antibacterial potential by improving the level of LZM at T1 and T2, although COS exerted a better effect than β-glucan. LZM is an important defense factor involved in non-specific immunity because it kills pathogens by hydrolyzing the bacterial cell wall. Its activity can be measured to reflect the degree of pathogen killing by phagocytic cells (40). Furthermore, bacteria enriched in the prebiotics group showed a significant positive correlation with the activity of LZM, including *Romboutsia*, *Cellulosilyticum*, and *Paraclostridium*. Hence, the two prebiotics could modulate the host’s immune response by altering the gut microbiome. In addition, the two prebiotics showed excellent antioxidant capacity, improving the activities of liver T-AOC and SOD and serum CAT and GSH-Px, with COS showing a better effect than β-glucan. GSH-dependent enzymes such as SOD, CAT, and GSH-Px participate in the scavenging of reactive oxygen species. GSH is the most important antioxidant molecule in the intracellular environment, and it can react with H_2_O_2_ or lipid peroxides to neutralize these molecules (41). Chang et al. demonstrated that COS could improve SOD and GSH-Px activities in the muscle tissue of broilers (42). He et al. revealed that dietary supplementation with 200 mg/kg yeast β-glucan can markedly increase CAT, SOD, and T-AOC activities in the skeletal muscle of finishing pigs (43). Further, all these antioxidant enzymes showed a close correlation with differential bacterial genera, especially in the liver. This was consistent with the fact that the liver is the most responsive tissue to abiotic stress and provides high constitutive protection against oxidative damage (44). Interestingly, we found that β-glucan improves the activity of ALP in the liver. Studies have shown that ALP plays a role in non-specific immunity in aquatic animals (45) and usually acts as a natural anti-inflammatory agent to reduce intestinal inflammation and alleviate associated insulin resistance (46). The increased activity of ALP demonstrated that lipid metabolism was enhanced in turtles with dietary β-glucan supplementation, suggesting that the prebiotics enhanced lipid metabolism in turtles and promoted the abundance of related bacteria, such as *Helicobacter* and *Mycoplasma*. Taken together, the findings show that COS and β-glucan can improve the immune response and antioxidant activity in both the liver and serum by regulating the gut microbiome.

The findings from the present study demonstrated that COS and β-glucan improve the content of total amino acids, essential amino acids, and umami amino acids in the hind leg muscles of turtles, thus improving meat quality. Further, β-glucan has a better effect than COS. The umami flavor of animal proteins is mainly determined by the content and composition of amino acids such as Glu, Asp, Ala, and Gly (47). Indeed, COS and β-glucan significantly improved the levels of these four umami amino acids, and 1% β-glucan increased the content of umami amino acids by 11.6%. COS has been reported to reduce abdominal fat and improve meat quality in broiler chickens (48). β-glucan has also been discovered to increase the content of intramuscular fat and change the proportion of saturated and unsaturated fatty acids in finishing pigs (49). However, this study provides the earliest evidence that COS and β-glucan can improve meat quality in turtles. Moreover, seven genera showed close correlations with these nutritional components. Further, *Clostridium_sensu_stricto_13* was significantly associated with all umami amino acids and some essential amino acids, strongly indicating its key role in improving meat quality and warranting further investigation.

The environmental impact of COS and β-glucan was tested. These prebiotics virtually changed the water quality in the paddy field farming system. COS and β-glucan both reduced the pH and sulfide content of culture water. Interestingly, 0.5% COS decreased the level of ammonia nitrogen, and 0.5% β-glucan decreased the level of nitrite. Previous studies have confirmed that reducing the pH, ammonia, and nitrite levels improves water quality (50). Probiotics, such as *Bacillus* spp., are usually used to improve water quality and growth performance. However, the administration of prebiotics as water quality regulators remains rare (51). Further analysis showed that COS and β-glucan changed the microbial community structure of culture water and sediment of paddy fields. Proteobacteria, Bacteroidota, and Actinobacteria — the dominant bacterial groups present in the culture water and sediment environment (52). The bacterial diversity of sediment was increased after prebiotic treatment in sediments, and the abundance of *Bacillus* and *Clostridium_sensu_stricto_1* were both increased in the water and sediment environment. It has been reported that some *Bacillus* spp. were capable to produce bioactive metabolites with antibacterial and antifungal properties against pathogens (53). Meanwhile, a recent study found that *Clostridium_sensu_stricto_1* is related to the expression of inflammation-related genes including REG3G, CCL8, and IDO1 (54), but its levels were decreased in our six prebiotics groups in the water and sediment samples of paddy field. Hence, COS and β-glucan could reshape the bacterial community of the culture water and improve water quality, even promoting the healthy growth of turtles.

One limitation of this study was that we could not study the effect of the two prebiotics on the growth performance of turtles. Because adult turtles are fed in paddy fields, it is difficult to catch and weigh them all. All the relationships between the phenotypes and gut microbiota were based on correlation studies. Hence, the effects and mechanisms through which the bacteria could potentially promote healthy growth, immune function enhancement, meat quality, and water quality were not identified. In the future, we will explore the effect of specific bacterial strains regulated by the prebiotics on growth traits through metagenomic sequencing and serum metabolomic analysis and provide more evidence regarding the application of prebiotics for promoting healthy growth in turtles. In addition, the synergistic or additive effect of different prebiotic combinations on the culture of Chinese soft-shelled turtle need to be considered. Except the analysis of liver and serum immunoenzyme activity, some important immune genes expression also need to be mensurated and demonstrate the effect of these prebiotic on immunity in the next step of our research.

## Conclusions

In summary, this study indicates that dietary supplementation with COS and β-glucan can reshape the colonic bacterial community structure and improve intestinal morphology in turtles during rice-turtle coculture. Furthermore, the administration of COS and β-glucan promoted antioxidant and immune enzyme activities in the liver and serum, with COS exerting stronger effects than β-glucan. These two prebiotics also improved the nutrient content of muscle tissue, particularly for essential and umami amino acids, and 1% β-glucan had a better effect than 1% COS. The observed improvements in meat quality and immunity were likely dependent on changes in the gut microbiota. The findings provide new evidence for supporting the application of COS and β-glucan as feed additives in turtle breeding. Moreover, COS and β-glucan improved the water quality of the culture system, which was accompanied by a decreased abundance of harmful bacteria associated with inflammation in the intestinal tract of turtles. This study not only provides a comprehensive understanding of the positive effects of COS and β-glucan on Chinese soft-shell turtles, but also promotes the application of prebiotics in aquaculture.

## Data availability statement

All 16S rRNA gene sequencing data were deposited in the National Center for Biotechnology Information sequence read archive (SRA) repository, accession number PRJNA974559 at https://www.ncbi.nlm.nih.gov/bioproject/PRJNA974559/.

## Ethics statement

The animal study was approved by The Institution of Animal Care and Use Committee (ACUC) of Zhejiang Academy of Agricultural Sciences. The study was conducted in accordance with the local legislation and institutional requirements.

## Author contributions

HF: Formal Analysis, Methodology, Software, Visualization, Writing – original draft. MQ: Data curation, Investigation, Validation, Writing – original draft. QY: Methodology, Project administration, Resources, Writing – original draft. ML: Methodology, Project administration, Resources, Writing – original draft. GY: Data curation, Investigation, Resources, Validation, Writing – original draft. WB: Methodology, Project administration, Resources, Writing – review & editing. TZ: Funding acquisition, Project administration, Supervision, Writing – review & editing. XP: Funding acquisition, Supervision, Writing – review & editing.

## Supplementary material

Supplementary Figure 1 Comparison of the diversity and bacterial composition of the gut microbiota at T2 based on 16S rRNA gene sequencing data. **(A)** Chao1 index. **(B)** Shannon index. **(C)** Principal coordinate analysis (PCoA) based on weighted UniFrac distances. **(D)** Relative abundance of the top 10 bacterial genera in the seven groups. **(E)** Differential bacterial genera identified among the seven groups using LEfSe analysis LDA score > 2. LDA, linear discriminant analysis. PERMANOVA, the permutational multivariate analysis of variance. one-way ANOVA, **P* < 0.05, ***P* < 0.01, and ****P* < 0.001.

Supplementary Figure 2 Colon morphology of *P. sinensis* at T2. **(A)** Full scans (×20). **(B)** Partial scans (×100).

Supplementary Figure 3 Correlations between differential bacterial genera and immune enzyme activity in the **(A)** liver and **(B)** serum of *P. sinensis*. Spearman’s rank correlation test, **P* < 0.05, ***P* < 0.01.

Supplementary Figure 4 Correlations between differential bacterial genera and muscle nutritional content in *P. sinensis*. Spearman’s rank correlation test, **P* < 0.05, ***P* < 0.01, and ****P* < 0.001.

Supplementary Figure 5 Predicted KEGG functional pathways of the gut microbiota enriched in each group at T1 **(A)** and their correlations with the relative abundance of differential genera **(B)**. Red arrows and lines indicate the main bacterial functions enriched in the COS and β-glucan groups, and green indicates the main bacterial functions enriched in the blank control group. Spearman’s rank correlation test, **P* < 0.05, ***P* < 0.01, and ****P* < 0.001.
