# Supplementary figures and images for: Effects of dietary chito-oligosaccharide and β-glucan on the water quality and gut microbiota, intestinal morphology, immune response, and meat quality of Chinese soft-shell turtle (Pelodiscus sinensis)
Source: Front Immunol. 2023 Oct 26;14:1266997. doi: 10.3389/fimmu.2023.1266997 (PMC10643201; doi:10.3389/fimmu.2023.1266997)

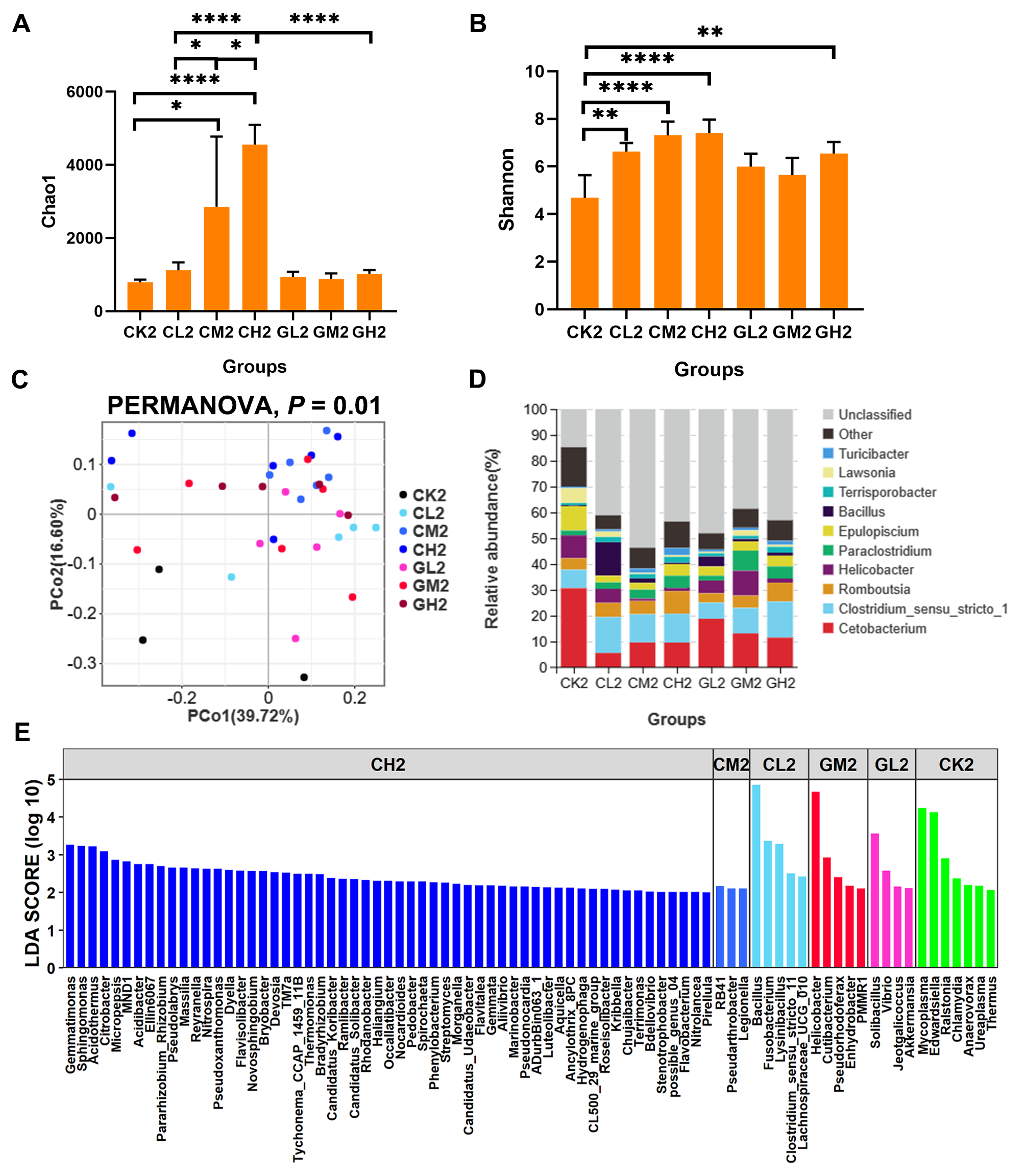

Supplement: Supplementary Figure 1 — Comparison of the diversity and bacterial composition of the gut microbiota at T2 based on 16S rRNA gene sequencing data. (A) Chao1 index. (B) Shannon index. (C) Principal coordinate analysis (PCoA) based on weighted UniFrac distances. (D) Relative abundance of the top 10 bacterial genera in the seven groups. (E) Differential bacterial genera identified among the seven groups using LEfSe analysis LDA score > 2. LDA, linear discriminant analysis. PERMANOVA, the permutational multivariate analysis of variance. one-way ANOVA, *P < 0.05, **P < 0.01, and ***P < 0.001. [file Image_1.tif]

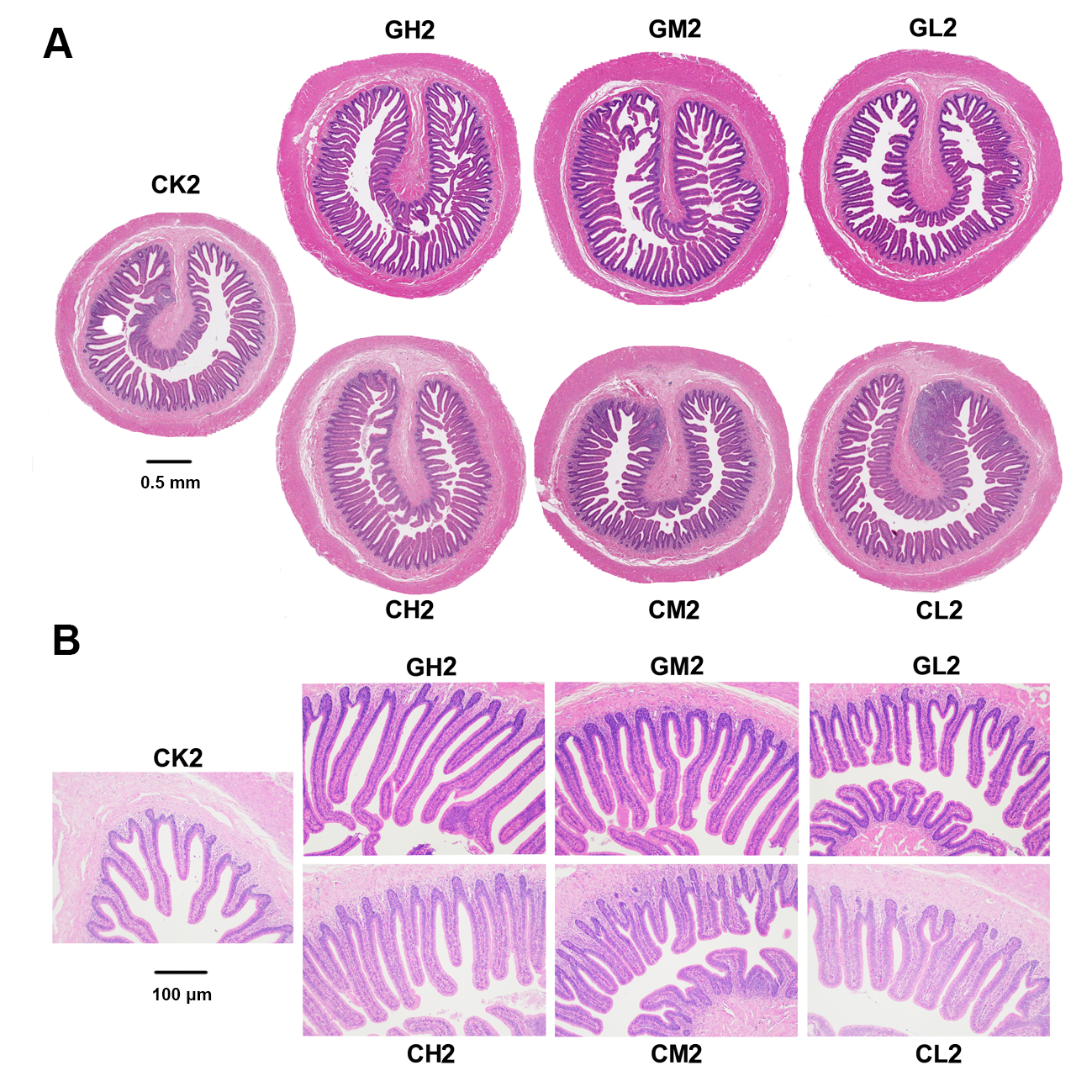

Supplement: Supplementary Figure 2 — Colon morphology of P. sinensis at T2. (A) Full scans (×20). (B) Partial scans (×100). [file Image_2.tif]

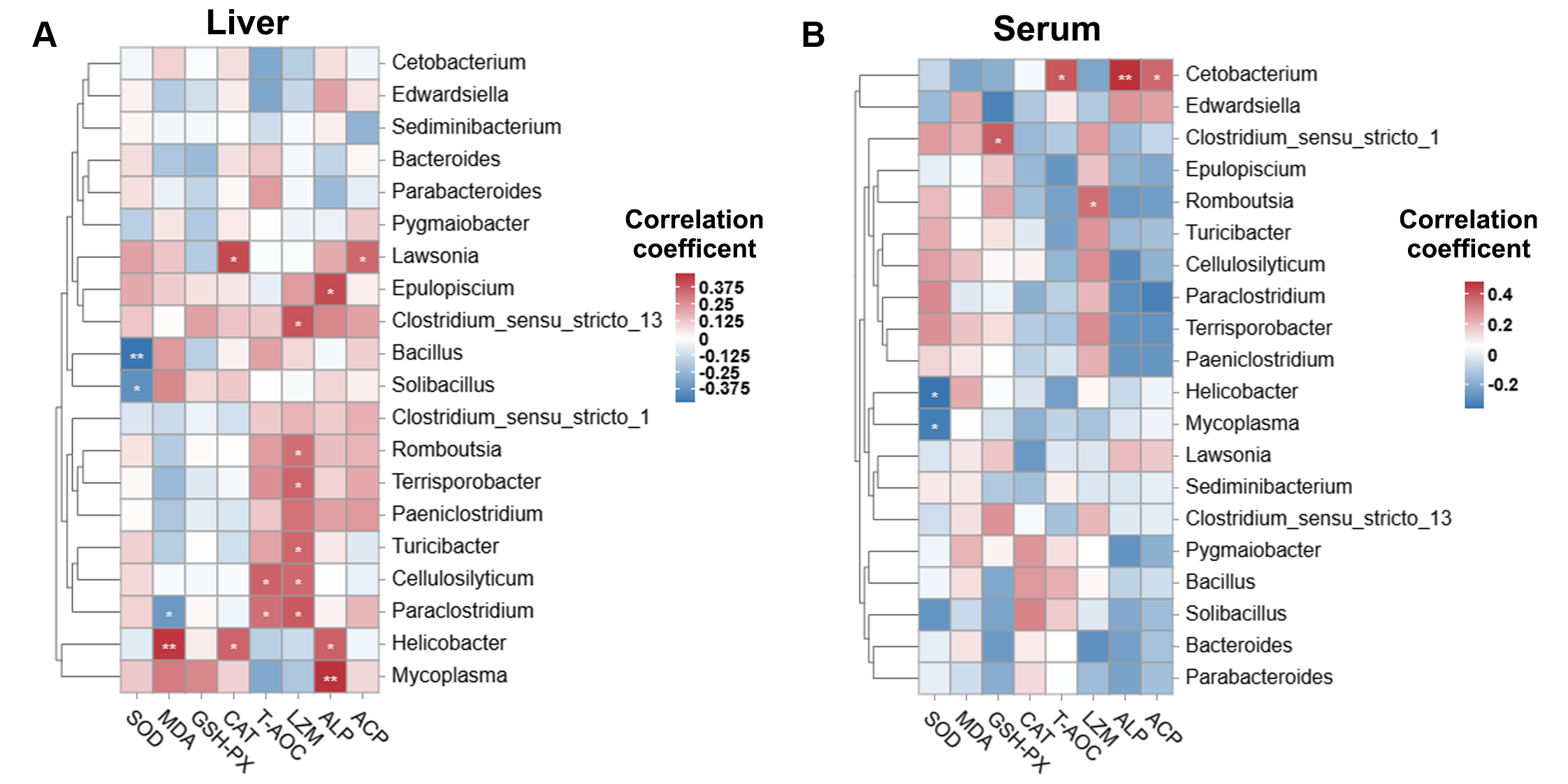

Supplement: Supplementary Figure 3 — Correlations between differential bacterial genera and immune enzyme activity in the (A) liver and (B) serum of P. sinensis. Spearman’s rank correlation test, *P < 0.05, **P < 0.01. [file Image_3.tif]

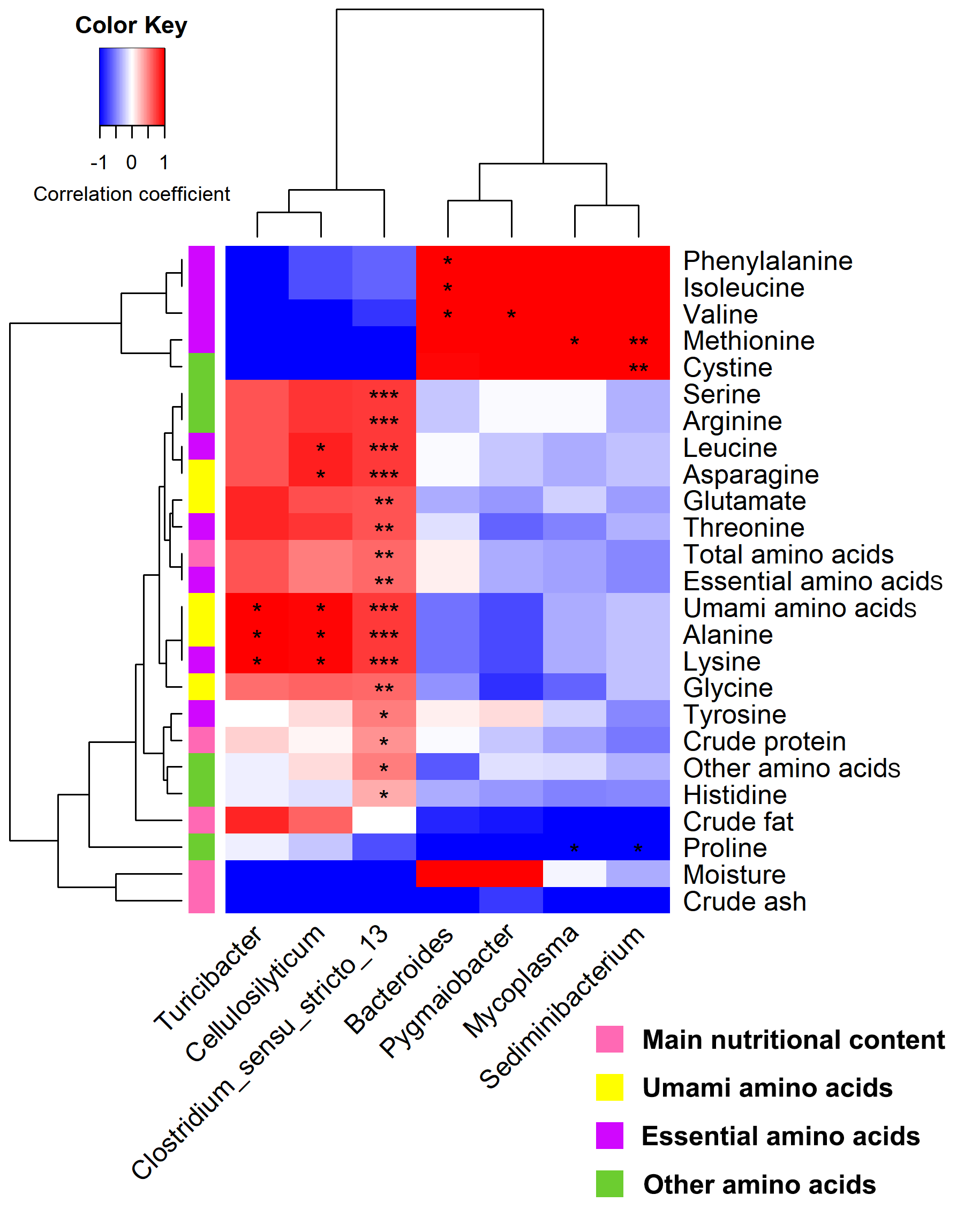

Supplement: Supplementary Figure 4 — Correlations between differential bacterial genera and muscle nutritional content in P. sinensis. Spearman’s rank correlation test, *P < 0.05, **P < 0.01, and ***P < 0.001. [file Image_4.tif]

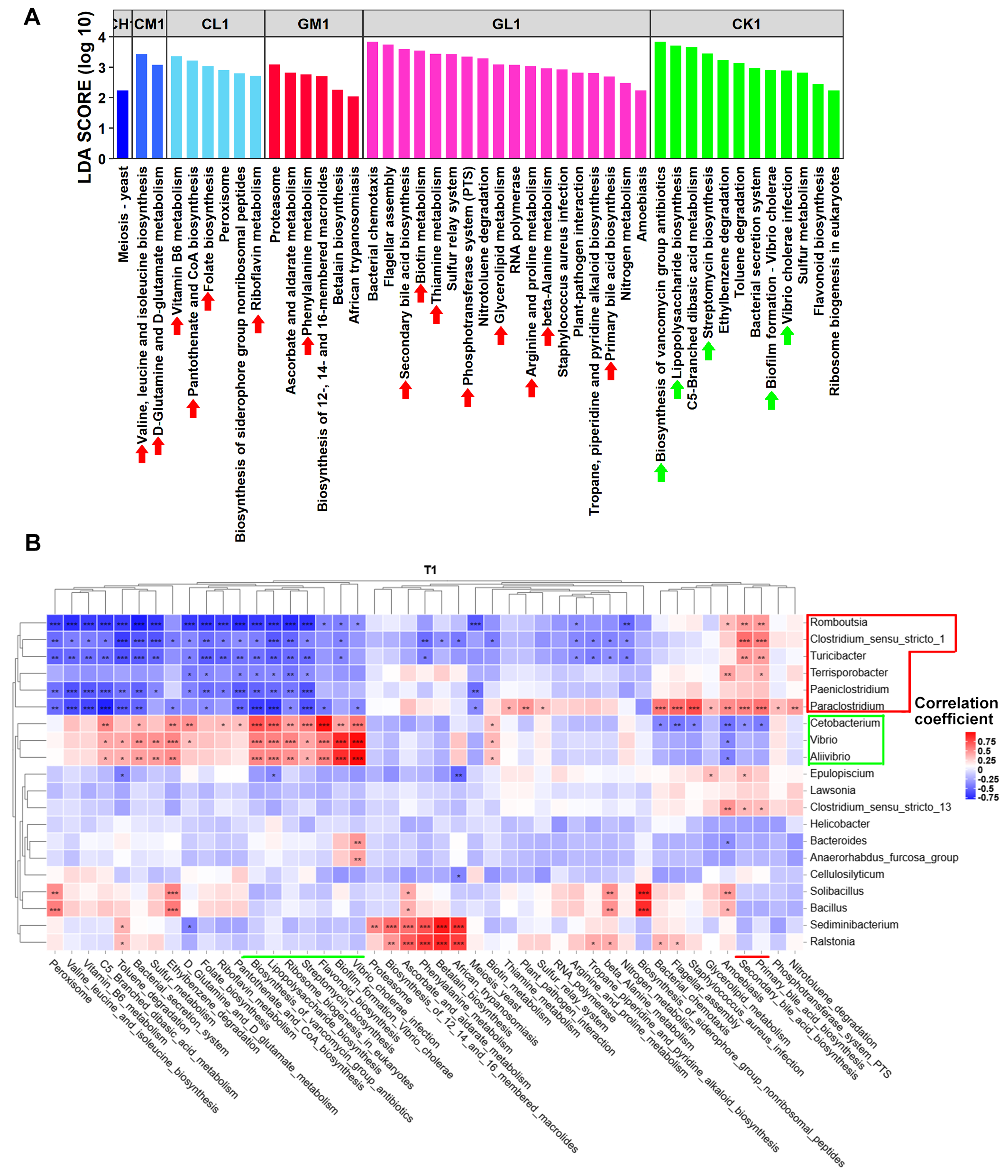

Supplement: Supplementary Figure 5 — Predicted KEGG functional pathways of the gut microbiota enriched in each group at T1 (A) and their correlations with the relative abundance of differential genera (B). Red arrows and lines indicate the main bacterial functions enriched in the COS and β-glucan groups, and green indicates the main bacterial functions enriched in the blank control group. Spearman’s rank correlation test, *P < 0.05, **P < 0.01, and ***P < 0.001. [file Image_5.tif]
